# Redetermination of 3-methyl­isoquinoline at 150 K

**DOI:** 10.1107/S1600536810039838

**Published:** 2010-10-09

**Authors:** Andrew D. Bond

**Affiliations:** a University of Southern Denmark, Department of Physics and Chemistry, Campusvej 55, 5230 Odense, Denmark

## Abstract

The structure of the title compound, C_19_H_9_O, has been redetermined at 150 K. The redetermination is of significantly higher precision than a previous room-temperature structure [Ribar *et al.* (1974 ▶). *Cryst. Struct. Commun.* **3**, 323–325]. The C—N bond lengths for this redetermination are much closer to those observed in comparable structures, and the orientation of the methyl group with respect to the isoquinoline plane is clarified. Inter­molecular weak C—H⋯N contacts are present in the crystal.

## Related literature

For the structure at room temperature, see: Ribar *et al.* (1974 ▶). For the structure of the parent compound isoquinoline, see: Hensen *et al.* (1999 ▶). The C—N bond length in the structure of Ribar *et al.* (1974 ▶) clearly lies outside of the main distribution for 19 relevant structural fragments in the Cambridge Structural Database, being the second shortest bond in the sample [one shorter bond exists for refcode SAKCIQ, but this structure has *R*1 = 14.2% (Trumpp-Kallmeyer *et al.*, 1998 ▶)]. The corresponding C—N bond length in this redetermination lies exactly at the mean of the CSD sample.
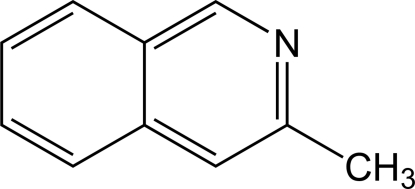


## Experimental

### 

#### Crystal data

C_10_H_9_N
*M* *_r_* = 143.18
Monoclinic, 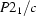

*a* = 6.1991 (4) Å
*b* = 7.4176 (6) Å
*c* = 16.5421 (12) Å
β = 93.438 (2)°
*V* = 759.28 (10) Å^3^
*Z* = 4
Mo *K*α radiation
μ = 0.07 mm^−1^
*T* = 150 K
0.25 × 0.15 × 0.12 mm

#### Data collection

Bruker–Nonius X8 APEXII CCD diffractometer
Absorption correction: multi-scan (*SADABS*; Sheldrick, 2003 ▶) *T*_min_ = 0.826, *T*_max_ = 0.991
9801 measured reflections
1844 independent reflections
1171 reflections with *I* > 2σ(*I*)
*R*_int_ = 0.034

#### Refinement

*R*[*F* ^2^ > 2σ(*F* ^2^)] = 0.042
*wR*(*F* ^2^) = 0.119
*S* = 1.06
1844 reflections
101 parameters
H-atom parameters constrained
Δρ_max_ = 0.24 e Å^−3^
Δρ_min_ = −0.20 e Å^−3^

### 

Data collection: *APEX2* (Bruker, 2004 ▶); cell refinement: *SAINT* (Bruker, 2003 ▶); data reduction: *SAINT*; program(s) used to solve structure: *SHELXTL* (Sheldrick, 2008 ▶); program(s) used to refine structure: *SHELXTL*; molecular graphics: *SHELXTL*; software used to prepare material for publication: *SHELXTL*.

## Supplementary Material

Crystal structure: contains datablocks global, I. DOI: 10.1107/S1600536810039838/hb5670sup1.cif

Structure factors: contains datablocks I. DOI: 10.1107/S1600536810039838/hb5670Isup2.hkl

Additional supplementary materials:  crystallographic information; 3D view; checkCIF report

## Figures and Tables

**Table 1:** Hydrogen-bond geometry (Å, °)

*D*—H⋯*A*	*D*—H	H⋯*A*	*D*⋯*A*	*D*—H⋯*A*
C5—H5*A*⋯N2^i^	0.95	2.88	3.6891 (14)	144
C6—H6*A*⋯N2^ii^	0.95	2.64	3.5813 (15)	170

Symmetry codes: (i) 
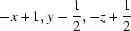
; (ii) 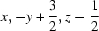.

**Fig. 1.**
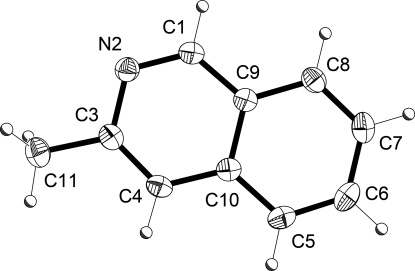
Molecular structure showing displacement ellipsoids at 50% probability for non-H atoms.

**Fig. 2.**
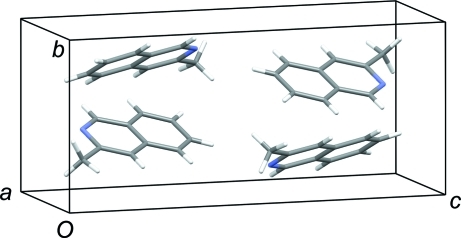
Unit-cell contents.

## supplementary crystallographic information

### Comment

The structure of 3-iso-methylquinoline at room temperature has been reported by Ribar *et al.* (1974). This redetermination at 150 K provides significantly improved precision, and more regular positions for the H atoms.

Considering the Cl1—N2 bond length: the CCDC *Mogul* package identifies 19 relevant structural fragments in the CSD, with a mean bond length of 1.314 (10) Å. The structure of Ribar *et al.* [C—N = 1.300 (5) Å] lies clearly outside of the main distribution, being the second shortest bond in the sample (one shorter bond of 1.292 Å exists for refcode SAKCIQ, but this structure has *R*1 = 14.2% (Trumpp-Kallmeyer *et al.*, 1998). By contrast, the C1—N2 bond length of 1.3144 (13) Å in this redetermination corresponds exactly with the mean value. Alternation is also more clearly seen for the bond lengths C5—C6, C6—C7 and C7—C8 (1.3649 (16), 1.4093 (16) and 1.3646 (15) Å, respectively), compared to the previous structure.

Concerning the H atoms, the orientation of the methyl group in particular is clarified: in the structure of Ribar *et al.*, the H—C(methyl)—H angles are irregular (range 94.8–112.8 °) and the orientation of the group is such that one C—H bond is twisted from the isoquinoline plane with a C—C—C(methyl)—H torsion angle *ca* 22 °. In the redetermination, the refined orientation of the methyl group places one C—H bond much more clearly in the isoquinoline plane (torsion angle 5.8 (1) °). This also has an influence on the geometry observed for the intermolecular contact between the methyl group and a neighbouring isoquinoline molecule. In the redetermination, atom H11B lies over the centroid of the C5—C10 ring with H11B···*Cg* = 2.95 Å and C11—H11B···*Cg* = 131.9 Å.

### Experimental

The colourless block of (I) used for structure determination was taken directly from the sample as supplied by Aldrich Chemical Company.

### Refinement

H atoms bound to C(*sp*^2^) were positioned geometrically with C—H = 0.95 Å and refined as riding with *U*_iso_(H) = 1.2 *U*_eq_(C). The H atoms of the methyl group were positioned with C—H = 0.98 Å and refined as riding with *U*_iso_(H) = 1.5 *U*_eq_(C), and with rotation about the local 3-fold axis.

### Crystal data

**Table:**

C_10_H_9_N	*F*(000) = 304
*M**_r_* = 143.18	*D*_x_ = 1.253 Mg m^−^^3^
Monoclinic, *P*2_1_/*c*	Melting point = 336–338 K
Hall symbol: -P 2ybc	Mo *K*α radiation, λ = 0.71073 Å
*a* = 6.1991 (4) Å	Cell parameters from 1780 reflections
*b* = 7.4176 (6) Å	θ = 2.5–25.4°
*c* = 16.5421 (12) Å	µ = 0.07 mm^−^^1^
β = 93.438 (2)°	*T* = 150 K
*V* = 759.28 (10) Å^3^	Block, colourless
*Z* = 4	0.25 × 0.15 × 0.12 mm

### Data collection

**Table:**

Bruker–Nonius X8 APEXII CCD diffractometer	1844 independent reflections
Radiation source: fine-focus sealed tube	1171 reflections with *I* > 2σ(*I*)
graphite	*R*_int_ = 0.034
thin–slice ω and φ scans	θ_max_ = 28.4°, θ_min_ = 3.7°
Absorption correction: multi-scan (*SADABS*; Sheldrick, 2003)	*h* = −7→8
*T*_min_ = 0.826, *T*_max_ = 0.991	*k* = −9→9
9801 measured reflections	*l* = −21→20

### Refinement

**Table:**

Refinement on *F*^2^	Primary atom site location: structure-invariant direct methods
Least-squares matrix: full	Secondary atom site location: difference Fourier map
*R*[*F*^2^ > 2σ(*F*^2^)] = 0.042	Hydrogen site location: inferred from neighbouring sites
*wR*(*F*^2^) = 0.119	H-atom parameters constrained
*S* = 1.06	*w* = 1/[σ^2^(*F*_o_^2^) + (0.0637*P*)^2^] where *P* = (*F*_o_^2^ + 2*F*_c_^2^)/3
1844 reflections	(Δ/σ)_max_ < 0.001
101 parameters	Δρ_max_ = 0.24 e Å^−^^3^
0 restraints	Δρ_min_ = −0.20 e Å^−^^3^

### Special details

**Table:**

Geometry. All e.s.d.'s (except the e.s.d. in the dihedral angle between two l.s. planes) are estimated using the full covariance matrix. The cell e.s.d.'s are taken into account individually in the estimation of e.s.d.'s in distances, angles and torsion angles; correlations between e.s.d.'s in cell parameters are only used when they are defined by crystal symmetry. An approximate (isotropic) treatment of cell e.s.d.'s is used for estimating e.s.d.'s involving l.s. planes.
Refinement. Refinement of *F*^2^ against ALL reflections. The weighted *R*-factor *wR* and goodness of fit *S* are based on *F*^2^, conventional *R*-factors *R* are based on *F*, with *F* set to zero for negative *F*^2^. The threshold expression of *F*^2^ > σ(*F*^2^) is used only for calculating *R*-factors(gt) *etc*. and is not relevant to the choice of reflections for refinement. *R*-factors based on *F*^2^ are statistically about twice as large as those based on *F*, and *R*- factors based on ALL data will be even larger.

### Fractional atomic coordinates and isotropic or equivalent isotropic displacement parameters (Å^2^)

**Table:**

	*x*	*y*	*z*	*U*_iso_*/*U*_eq_	
C1	0.11212 (16)	0.91552 (15)	0.31390 (6)	0.0232 (3)	
H1A	−0.0145	0.9742	0.3301	0.028*	
N2	0.26422 (14)	0.87917 (12)	0.37033 (5)	0.0247 (3)	
C3	0.44782 (16)	0.79419 (15)	0.34720 (7)	0.0234 (3)	
C4	0.47647 (16)	0.75059 (15)	0.26814 (7)	0.0232 (3)	
H4A	0.6070	0.6944	0.2544	0.028*	
C5	0.32884 (18)	0.74255 (15)	0.12395 (7)	0.0257 (3)	
H5A	0.4564	0.6873	0.1066	0.031*	
C6	0.16084 (19)	0.77766 (16)	0.06908 (7)	0.0294 (3)	
H6A	0.1709	0.7434	0.0141	0.035*	
C7	−0.02738 (18)	0.86413 (16)	0.09309 (7)	0.0289 (3)	
H7A	−0.1420	0.8894	0.0540	0.035*	
C8	−0.04638 (16)	0.91186 (15)	0.17202 (7)	0.0247 (3)	
H8A	−0.1734	0.9708	0.1877	0.030*	
C9	0.12341 (16)	0.87349 (14)	0.23056 (6)	0.0205 (3)	
C10	0.31404 (16)	0.78813 (14)	0.20670 (7)	0.0209 (3)	
C11	0.61079 (18)	0.74985 (17)	0.41473 (7)	0.0317 (3)	
H11A	0.6495	0.8598	0.4451	0.048*	
H11B	0.7403	0.6991	0.3923	0.048*	
H11C	0.5493	0.6617	0.4510	0.048*	

### Atomic displacement parameters (Å^2^)

**Table:**

	*U*^11^	*U*^22^	*U*^33^	*U*^12^	*U*^13^	*U*^23^
C1	0.0222 (6)	0.0219 (7)	0.0259 (6)	0.0014 (5)	0.0044 (5)	0.0006 (5)
N2	0.0262 (5)	0.0249 (6)	0.0232 (5)	0.0014 (4)	0.0022 (4)	0.0010 (4)
C3	0.0235 (6)	0.0196 (7)	0.0271 (7)	−0.0011 (4)	0.0003 (5)	0.0040 (5)
C4	0.0198 (5)	0.0212 (6)	0.0289 (7)	0.0011 (4)	0.0050 (5)	0.0025 (5)
C5	0.0300 (6)	0.0221 (6)	0.0258 (7)	0.0000 (5)	0.0089 (5)	−0.0002 (5)
C6	0.0402 (7)	0.0277 (7)	0.0206 (6)	−0.0059 (5)	0.0043 (5)	0.0007 (5)
C7	0.0287 (6)	0.0299 (7)	0.0274 (7)	−0.0049 (5)	−0.0050 (5)	0.0048 (5)
C8	0.0216 (6)	0.0233 (7)	0.0290 (7)	−0.0006 (4)	0.0001 (5)	0.0028 (5)
C9	0.0211 (5)	0.0172 (6)	0.0233 (6)	−0.0019 (4)	0.0028 (4)	0.0021 (5)
C10	0.0223 (6)	0.0173 (6)	0.0235 (6)	−0.0028 (4)	0.0042 (4)	0.0016 (5)
C11	0.0303 (6)	0.0335 (8)	0.0307 (7)	0.0015 (5)	−0.0040 (5)	0.0053 (6)

### Geometric parameters (Å, °)

**Table:**

C1—N2	1.3144 (13)	C6—C7	1.4093 (16)
C1—C9	1.4194 (15)	C6—H6A	0.950
C1—H1A	0.950	C7—C8	1.3646 (15)
N2—C3	1.3753 (13)	C7—H7A	0.950
C3—C4	1.3690 (15)	C8—C9	1.4157 (14)
C3—C11	1.4971 (15)	C8—H8A	0.950
C4—C10	1.4148 (15)	C9—C10	1.4174 (15)
C4—H4A	0.950	C11—H11A	0.980
C5—C6	1.3649 (16)	C11—H11B	0.980
C5—C10	1.4184 (16)	C11—H11C	0.980
C5—H5A	0.950		
			
N2—C1—C9	124.73 (10)	C8—C7—H7A	119.7
N2—C1—H1A	117.6	C6—C7—H7A	119.7
C9—C1—H1A	117.6	C7—C8—C9	119.93 (10)
C1—N2—C3	117.83 (9)	C7—C8—H8A	120.0
C4—C3—N2	122.08 (10)	C9—C8—H8A	120.0
C4—C3—C11	122.72 (10)	C8—C9—C10	119.83 (10)
N2—C3—C11	115.20 (10)	C8—C9—C1	122.84 (10)
C3—C4—C10	120.81 (10)	C10—C9—C1	117.33 (10)
C3—C4—H4A	119.6	C4—C10—C9	117.21 (10)
C10—C4—H4A	119.6	C4—C10—C5	124.19 (10)
C6—C5—C10	120.36 (10)	C9—C10—C5	118.59 (10)
C6—C5—H5A	119.8	C3—C11—H11A	109.5
C10—C5—H5A	119.8	C3—C11—H11B	109.5
C5—C6—C7	120.73 (11)	H11A—C11—H11B	109.5
C5—C6—H6A	119.6	C3—C11—H11C	109.5
C7—C6—H6A	119.6	H11A—C11—H11C	109.5
C8—C7—C6	120.54 (11)	H11B—C11—H11C	109.5
			
C9—C1—N2—C3	−0.04 (17)	N2—C1—C9—C8	178.66 (10)
C1—N2—C3—C4	1.19 (16)	N2—C1—C9—C10	−0.81 (17)
C1—N2—C3—C11	−177.61 (9)	C3—C4—C10—C9	0.55 (16)
N2—C3—C4—C10	−1.46 (17)	C3—C4—C10—C5	−178.11 (10)
C11—C3—C4—C10	177.24 (10)	C8—C9—C10—C4	−178.97 (9)
C10—C5—C6—C7	1.80 (17)	C1—C9—C10—C4	0.51 (15)
C5—C6—C7—C8	−1.03 (18)	C8—C9—C10—C5	−0.23 (16)
C6—C7—C8—C9	−0.38 (17)	C1—C9—C10—C5	179.26 (9)
C7—C8—C9—C10	0.99 (16)	C6—C5—C10—C4	177.49 (10)
C7—C8—C9—C1	−178.46 (10)	C6—C5—C10—C9	−1.16 (16)

### Hydrogen-bond geometry (Å, °)

**Table:**

*D*—H···*A*	*D*—H	H···*A*	*D*···*A*	*D*—H···*A*
C5—H5A···N2^i^	0.95	2.88	3.6891 (14)	144
C6—H6A···N2^ii^	0.95	2.64	3.5813 (15)	170

Symmetry codes: (i) −*x*+1, *y*−1/2, −*z*+1/2; (ii) *x*, −*y*+3/2, *z*−1/2.
